# Lymphoproliferative Disorders Mimicked by Tuberculosis: A Retrospective Study on Lateral Flow Urine Lipoarabinomannan (LF-ULAM) Limitations

**DOI:** 10.7759/cureus.85105

**Published:** 2025-05-30

**Authors:** Arthur E McKinnon, Yencke L Spammer, Sithembiso A Sikhosana

**Affiliations:** 1 Internal Medicine, Rob Ferreira Tertiary Hospital, Nelspruit, ZAF

**Keywords:** chronic lymphoproliferative disorders, classic hodgkin lymphoma, disseminated tuberculosis, extra-pulmonary tuberculosis (eptb), kaposi sarcoma, lateral flow urine lipoarabinomannan assay, lymphadenopathy, multicentric castleman disease, non-hodgins lymphoma, people living with hiv/aids

## Abstract

Introduction

The human immunodeficiency virus (HIV) remains South Africa's (SA) largest epidemic. Furthermore, tuberculosis (TB) continues to be the leading cause of death in SA. People living with HIV/AIDS (PLHA) are at increased risk of both TB and lymphoproliferative disorders (LPD). PLHA commonly manifest lymphadenopathy as part of their disease spectrum, with extra-pulmonary TB being the most common cause. Local guidelines recommend lateral flow urine lipoarabinomannan (LF-ULAM) assay testing for all PLHA admitted to the hospital. The LF-ULAM assay is therefore widely used in SA. The LF-ULAM assay does not provide definitive confirmation of the cause of lymphadenopathy. In resource-limited settings, TB can impetuously be attributed as the sole cause of lymphadenopathy in many PLHA with positive LF-ULAM assays. Misdiagnosis of LPD as TB can lead to catastrophic patient outcomes. Therefore, initial correct diagnosis is of utmost importance.

Aims and objectives

The aims and objectives of this study are to identify the etiology of lymphadenopathy in PLHA with positive LF-ULAM assays and to highlight the need for a high index of suspicion of LPD in PLHA with lymphadenopathy.

Materials and methods

A retrospective census study was carried out at a tertiary hospital in a rural province of SA. A total of 13 PLHA with lymphadenopathy, diagnosed with disseminated TB by LF-ULAM assay, were identified for this study. Eligible participants were identified using medical records from the medical wards. All PLHA included had generalized lymphadenopathy diagnosed by clinical palpation. Histopathologists were unblinded to LF-ULAM results. The results of TB investigations, excisional lymph node biopsies, and trephine biopsies from these patients were analyzed to confirm the etiology of their lymphadenopathy.

Results

A total of 13 PLHA were included in this study. All patients were initiated on anti-tuberculous treatment (ATT) following LF-ULAM assay positivity. The majority of cases (n = 9/13; 69%) in this small cohort had histological confirmation of an LPD. More than half of the cases (n = 7/13; 53%) were confirmed to have mycobacterial disease by means of either histology, culture, or TB nucleic acid amplification test (NAAT).

Conclusion

Histological confirmation of the etiology of lymphadenopathy is crucial in PLHA to differentiate between LPD and TB. Empiric TB treatment without appropriate confirmation of TB can lead to worse patient outcomes and significantly delay oncological care for PLHA. We recommend expanding biopsy access in resource-limited settings to prevent diagnostic delays and improve patient outcomes.

## Introduction

The human immunodeficiency virus (HIV) remains South Africa’s (SA) largest epidemic, with a prevalence of approximately 7.8 million people reported in a recent analysis. Furthermore, SA has the highest burden of advanced HIV disease (AHD), defined as a World Health Organization (WHO) stage 3/4 event or a cluster of differentiation 4 (CD4) of <200 cells/µL in adolescents or adults, in sub-Saharan Africa [1].

People living with HIV/AIDS (PLHA) are at increased risk of lymphoma and other lymphoproliferative disorders (LPD) [2]. HIV infection predisposes to cancer development due to impaired cellular immunity [3-5]. Tuberculosis (TB) and LPD can present in similar ways. Both conditions can co-exist in the same patient, confounding unequivocal diagnosis [6,7]. With antiretroviral therapy (ART) now readily available and the average lifespan of PLHA increasing, the prevalence of malignancies in the HIV population has increased. As a result, malignancies are significantly adding to the morbidity and mortality of these patients [4,8,9].

PLHA frequently manifest lymphadenopathy as part of their disease spectrum. The plethora of causes includes infections such as TB, atypical mycobacteria, toxoplasmosis, histoplasmosis, cryptococcosis, cytomegalovirus, and many more. PLHA are more prone to developing malignancies such as non-Hodgkin lymphoma (NHL), Hodgkin lymphoma (HL), and other LPD that commonly present with lymphadenopathy [10].

In 2014, the WHO advocated for the development of a highly specific and “rapid biomarker-based, non-sputum test capable of detecting all forms of TB by identifying characteristic biomarkers”. The latter included the aim to diagnose active TB and allow same-day test and treatment. Lateral flow urine lipoarabinomannan (LF-ULAM) assays test for the presence of lipoarabinomannan antigen in the urine. This antigen is present in active TB. The LF-ULAM is a convenient test for PLHA and suspected extra-pulmonary TB, such as those with lymphadenopathy, in whom traditional sputum-based assays have proven to be less sensitive [11]. It is currently part of the national guidelines in SA to perform an LF-ULAM assay on all severely ill PLHA admitted to a hospital, regardless of their clinical stage or CD4 count [12].

The aims and objectives of this study are to identify the etiology of lymphadenopathy in PLHA with positive LF-ULAM assays and to highlight the need for a high index of suspicion of LPD in PLHA with lymphadenopathy.

## Materials and methods

Data source and study design

A retrospective census study of the results of excisional lymph node and trephine biopsies in PLHA and positive LF-ULAM assays was carried out at a tertiary hospital in a rural province of SA. The hospital functions as a referral facility for the neighboring hospitals and clinics. Eligible cases were collected over the period from January 1, 2023, to December 31, 2023. The medical records of 13 patients in total were analyzed. All medical records were paper-based. All patient tissue samples were excisional lymph nodes and trephine biopsies.

Inclusion and exclusion criteria

Inclusion criteria were as follows: adult (age >18) PLHA with a positive LF-ULAM assay and a clinical history of lymphadenopathy, who have had a lymph node biopsy with or without trephine biopsy performed. 

For the exclusion criteria, exclusion occurred in the instance of any HIV negative case. PLHA with lymphadenopathy and a negative LF-ULAM assay in this period were not included in the cohort. Additionally, PLHA without lymphadenopathy and a positive LF-ULAM were not included in this cohort. PLHA with lymphadenopathy and a positive LF-ULAM assay in whom no tissue sample was taken were not included in this cohort. No patients < 18 years of age were included in this cohort.

Data collection and variables of interest

A data extraction template was used to collect data from eligible patient files. This template included a table with labels for LF ULAM results, demographic characteristics such as age, ethnicity, gender, HIV test results, the use of ART, CD4 count, tissue samples collected, and their sample sites, and all investigations done to examine for TB. The point-of-care Alere LF-ULAM assay was used to test for disseminated TB in all patients included in this study. A written record is kept of each LF-ULAM result in the medical wards. This record was utilized to identify all patients with positive LF-ULAM assays over the study period. TB investigations available in the medical records results charts were also analyzed and recorded on the data collection template. Thereafter, the medical records of patients confirmed to have HIV infection were isolated. Finally, the medical records of all eligible patients were scrutinized to identify patients noted to have lymphadenopathy, and who had an excisional lymph node biopsy with or without a trephine biopsy performed. All excisional lymph nodes and trephine biopsies were performed as in-hospital bedside procedures. All PLHA included had generalized lymphadenopathy diagnosed by clinical palpation. 

The primary aim was to identify the etiology of lymphadenopathy in PLHA and positive LF-ULAM assays. The results of all specimens sent for histological examination are recorded in the written medical records of each patient. The primary aim was achieved by analyzing the histological results of all eligible cases. Histopathologists were unblinded to LF-ULAM results. The results of biospecimen data extrapolated from the medical records were presented as percentages of the total number of patients.

Data analysis and statistical methods

Descriptive statistics were used to summarize baseline demographic and clinical variables. Normality of continuous variables (e.g., age, CD4 count) was assessed using the Shapiro-Wilk test. Means and standard deviations were used for normally distributed data. Medians and interquartile ranges were used for skewed data (e.g., waiting time for histological results). Due to the small sample size (n = 13), inferential statistical testing was not conducted. Convenience sampling was used for collecting patient data from medical wards. Quality assurance measures, including checking duplicate records and only using records that were complete and already clinically interpreted, were implemented. All analyses were performed using Microsoft Excel (Microsoft® Corp., Redmond, WA).

Ethical considerations

Ethics clearance was sought from and approved by the Mpumalanga Provincial Health Research Ethics Committee International Review Board (MPHREC IRB) (approval reference number: MP_202404_006). A waiver of informed consent was granted. Patient data and biospecimen data were anonymized and scrutinized by making use of Microsoft Excel on a password-protected computer stored in a secure room at the data collection facility. The research team had sole access to this room. The results were summarized and presented using descriptive statistical methods. A copy of the research study’s findings was sent to the MPHREC-IRB and the hospital’s clinical manager. 

## Results

Demographic characteristics, such as age, ethnicity, and gender, are presented in Table 1. Most of the cases were men (n = 10/13, 77%), and the minority were women (n = 3/13, 23%). Most of the cases (n = 8/13, 62%) were in the age group of 31-45 years, with the mean age being 43 years (SD = 11). The mean age of cases diagnosed with LPD was 41 years (SD = 10). All the cases were Black African.

**Table 1:** Demographic characteristics B: Black; F: female; M: male

Case	Age	Gender	Ethnicity
1	62	M	B
2	52	M	B
3	35	M	B
4	41	M	B
5	52	M	B
6	65	M	B
7	48	M	B
8	48	M	B
9	31	M	B
10	35	F	B
11	34	F	B
12	38	M	B
13	36	F	B

All patients in this cohort presented with B symptoms, including weight loss (more than 10% of normal body weight), and recurrent fever. Three of 13 patients presented with drenching night sweats as well. All patients had generalized lymphadenopathy. Lymphadenopathy was diagnosed by means of clinical palpation in all patients. The aforementioned refers to enlarged lymph nodes in two or more non-contiguous regions. 

The minority (n = 3/13, 23%) of patients were on ART when they presented to our facility (Table 2). The mean CD4 count of our cohort was 278 cells/µL (SD = 273) (Table 2). The mean CD4 count was lowest in patients confirmed to have TB by other means than the LF-ULAM assay, at 106 cells/µL (SD = 54). The mean CD4 count in cases with an LPD was 339 cells/µL (SD = 294). A large portion of the patients in this cohort had AHD (n = 7/13, 53%). Most of the lymph node samples were axillary nodes (n = 9/13, 69%), cervical nodes made up (n = 3/13, 23%), and inguinal nodes (n = 1/13, 8%) (Table 3). Three of 13 patients (23%) had a bone marrow aspirate and trephine biopsy as an additional sample. 

**Table 2:** Tuberculosis and HIV related laboratory investigations ADA: adenosine deaminase; AFB: acid fast bacilli; CD4: cluster of differentiation 4; CSF: cerebrospinal fluid; HIV: human immunodeficiency virus; LF-ULAM: lateral flow urine lipoarabinomannan; NAAT: nucleic acid amplification test; N/A: not applicable; TB: tuberculosis

Case	ULAM	TB NAAT	AFB	TB culture	Other	CD4	HIV	ART
1	+ve	Sputum -ve	Sputum -ve	Sputum -ve	Lymph node biopsy, suggestive of TB	122 cells/µL	+ve	No
2	+ve	Sputum -ve	Sputum -ve	Sputum -ve	N/A	344 cells/µL	+ve	No
3	+ve	CSF +ve	CSF +ve	CSF +ve	Lymph node biopsy, suggestive of TB	105 cells/µL	+ve	No
4	+ve	Sputum -ve	Sputum -ve	Sputum -ve	N/A	329 cells/µL	+ve	No
5	+ve	Sputum +ve	Sputum +ve	Sputum +ve	Lymph node biopsy, suggestive of TB	113 cells/µL	+ve	No
6	+ve	Sputum -ve	Sputum -ve	Sputum -ve	Pleural fluid ADA 73	150 cells/µL	+ve	No
7	+ve	Sputum -ve	Sputum -ve	Sputum -ve	N/A	946 cells/µL	+ve	No
8	+ve	Sputum -ve	Sputum -ve	Sputum -ve	N/A	153 cells/µL	+ve	Yes
9	+ve	Sputum -ve	Sputum -ve	Sputum -ve	Trephine biopsy, suggestive of TB	72 cells/µL	+ve	No
10	+ve	Sputum -ve	Sputum -ve	Sputum -ve	Lymph node biopsy, suggestive of TB	227 cells/µL	+ve	No
11	+ve	Sputum -ve	Sputum -ve	Sputum -ve	N/A	757 cells/µL	+ve	Yes
12	+ve	Sputum -ve	Sputum -ve	Sputum +ve	N/A	44 cells/µL	+ve	No
13	+ve	Sputum -ve	Sputum -ve	Sputum -ve	N/A	260 cells/µL	+ve	Yes

**Table 3:** Cytomorphology BMAT: bone marrow aspirate and trephine biopsy; CD4: cluster of differentiation 4; HHV-8: human herpes-virus 8; N/A: not applicable; PCR: polymerase chain reaction; TB: tuberculosis; ZN: Ziehl-Neelsen

Case	Lymph node cytomorphology	Site of lymph node biopsy	BMAT cytomorphology
1	Granulomatous inflammation with caseous necrosis, ZN +ve	Axillary	N/A
2	Mixed cellularity Hodgkin lymphoma	Axillary	N/A
3	Necrotizing granulomatous inflammation, ZN +ve	Axillary	N/A
4	Follicular lymphoma	Axillary	N/A
5	Granulomatous inflammation with caseous necrosis, ZN -ve. TB PCR +ve	Cervical	Overt hemophagocytosis
6	Diffuse large B-cell lymphoma	Cervical	N/A
7	Diffuse large B-cell lymphoma L and nodal Kaposi sarcoma	Axillary	N/A
8	Lymphoblasts present. Features of involvement in acute lymphoblastic leukemia	Axillary	Acute lymphoblastic leukemia with 91% blasts
9	Hodgkin lymphoma	Cervical	Involvement by HL. Granulomatous inflammation, ZN -ve
10	Granulomatous inflammation, ZN +ve	Axillary	N/A
11	Castleman’s disease - hyaline vascular subtype	Inguinal	N/A
12	High-grade B-cell lymphoma	Axillary	N/A
13	HHV-8-associated multicentric Castleman’s disease	Axillary	N/A

Table 2 presents the methods by which the diagnosis of TB was established in each case. All thirteen patients were initially diagnosed with disseminated TB by LF-ULAM on admission and promptly initiated on anti-tuberculous therapy (ATT). TB was confirmed with lymph node or trephine biopsy in five of 13 (38%) cases. Seven of 13 (53%) cases had TB confirmed by means of an additional test such as TB nucleic acid amplification test (NAAT), TB microscopy, TB culture, or suggestive features on histological examination. Six of 13 (46%) patients were diagnosed with TB solely by LF-ULAM assay. Our facility is not able to do TB or tissue culture on lymph node samples, and therefore, these results are absent from our investigations. 

The lymph node, bone marrow aspirate, and trephine biopsy cytomorphology of each case are presented in Table 3. The highest number of patient samples (n = 9/13, 69%) had histological confirmation of an LPD (Table 3). NHL was the most common LPD (n = 4/13, 31%), HL comprised 15% (n = 2/13), human herpesvirus-8-associated Castleman's disease accounted for 15% (n = 2/13), and acute B lymphoblastic leukemia accounted for 8% (n = 1/13). One patient fulfilled diagnostic criteria for hemophagocytic lymphohistiocytosis, and one patient had features suggestive of both diffuse large B-cell lymphoma and nodal Kaposi sarcoma in the same sample. Moreover, 38% (n = 5/13) of the patient samples revealed granulomatous inflammation, and 15% (n = 2/13) of the samples showed caseous necrosis. Mycobacterial disease was confirmed by Ziehl-Neelsen stains in 23% (n = 3/13) of patients and TB complex polymerase chain reaction in 8% (n = 1/13) of patients.

## Discussion

Key findings

This retrospective study included PLHA with lymphadenopathy and a positive LF-ULAM assay. All the patients included were initially diagnosed with disseminated TB by LF-ULAM assay and initiated on ATT. An alarming number of patient samples in this small cohort had histological proof of an LPD (n = 9/13, 69%) mimicked by TB. The most common LPD was NHL (n = 4/13, 31%). Thirty-eight percent of the lymph node and trephine samples had histological evidence suggestive of TB. The highest number of cases in this cohort was male (n = 10/13, 77%). Most of the excisional lymph node samples were axillary lymph nodes (n = 9/13, 69%).

Comparison to literature and implications

PLHA are at increased risk of LPD and TB, and both disease entities can exist concurrently in the same patient [6,7]. TB and lymphoma are among the greatest mimickers in medicine’s history, frequently found to be the culprit in the misdiagnosis of each other, as well as various other diseases. Additionally, their routinely shared symptomatology, clinical, and radiological features commonly confound unequivocal diagnosis [5,13].

The LF-ULAM provides valuable information on many patients’ clinical scenarios. However, LF-ULAM assays do not provide drug sensitivity or microbiological confirmation of disease. This is an important fact, and it is imperative that the treating physician continues to try to find an alternative diagnostic sample to assist with drug sensitivity and microbiological confirmation of TB, ideally, by means of culture, microscopy, or NAAT. The LF-ULAM's sensitivity and specificity are dependent on CD4 counts and whether patients are symptomatic. Sensitivity significantly declines when CD4 counts are >200 cells/µL to roughly 16%. In symptomatic patients with CD4 counts <200 cells/µL, the sensitivity is around 45%. In a recent review, specificity in symptomatic patients with CD4 > 200 cells/µL was 94%, and for those <200 cells/µL, it was 89%. Although the test remains very specific regardless of CD4 count, false negative results are significantly increased in patients with CD4 counts > 200 cells/µL [11,14].

In the current context of SA’s public health sector, there are severe resource constraints and a lack of excisional lymph node biopsy services at all centers. Therefore, this could result in cases of PLHA and generalized lymphadenopathy diagnosed with extra-pulmonary TB by LF-ULAM assay not being further investigated. The etiology of lymphadenopathy in these patients can impetuously be attributed solely to TB, leading to possible misdiagnosis of LPD as TB [5]. The study facility is in a rural province without any local pathology services. All tissue samples were sent away to a neighboring province. The average waiting time for histology results is roughly four weeks. The median waiting time for our cohort's results was five weeks. The prior statistic highlights the importance of a correct initial diagnosis. As TB is endemic in this setting, it is common practice to treat patients empirically with ATT and monitor for clinical response to treatment while awaiting tissue sample results. TB culture remains the gold standard for TB diagnosis, and it has been shown in a previous local study, by Puvaneswaran and Shoba, that misdiagnosis of lymphoma as TB can significantly impact patient care as well as mortality and morbidity. Their study found that SA patients diagnosed with lymphoma were on TB treatment for a median of five months before the correct diagnosis of lymphoma was made, with many of these patients not having proven TB [15,16]. Additionally, it has been highlighted in previous studies that 25-85% of lymphoma patients are on TB therapy at the time of diagnosis [3-5,15]. There are inherent dangers to empiric TB treatment. The former refers to ATT initiation without bacteriological confirmation. Although empiric TB treatment benefits patients who truly have TB, a randomized controlled trial has shown that it does not improve overall mortality in PLHA with AHD [17]. Furthermore, studies have found that empiric TB treatment may, in fact, increase mortality and morbidity when other etiologies are misdiagnosed as TB in PLHA [18,19]. There is a paucity of guidance regarding the appropriate time period to monitor for treatment response to ATT for tuberculous lymphadenitis. Some studies advise a period of two months, but report that there may be a paradoxical expansion of adenopathy in up to 20% of cases [20]. This retrospective study further amplifies the need for further investigation into the etiology of these patients’ lymphadenopathy in order not to unnecessarily delay their oncological care. 

Recommendations

Due to the common association of HIV and TB, together with more documented prevalence of LPD in PLHA, a high index of suspicion for concurrent and misleading presentations should be maintained in these patients [6,7,15]. Hickam’s dictum, the principle that a patient can have more than one disease at the same time, may be a more appropriate approach to PLHA with lymphadenopathy [21,22]. The findings of this study should not discourage physicians from performing LF-ULAM tests, as per national guidelines. However, these findings should alert physicians that PLHA may have more than one diagnosis and prompt further analyses. We recommend that bacteriological or histological confirmation of the cause of lymphadenopathy be sought in all patients with positive LF-ULAM assays. Lastly, we recommend that access to biopsy services in resource-limited settings be made a priority. It is this center’s current practice that any worsening in the condition of a patient on ATT should be regarded as significant and warrants investigation into alternative diagnoses to explain the patient's condition.

Limitations

There are potential limitations to the study design. Firstly, this study included a small cohort. However, the data support the need for diagnostic vigilance in PLHA with lymphadenopathy and positive LF-ULAM assays. The cohort size was influenced by the specific inclusion criteria of the study. This was a retrospective study, with a lack of inferential statistical analysis and clinical outcome data. Additionally, it was an unblinded study without any control groups. Future studies that include control groups and that are blinded may be able to draw more significant conclusions. The conclusions drawn pertain only to this specific group of PLHA. This was a single-center study. Larger trials involving multiple centers in their design will draw more significant conclusions. This study is specific to resource-limited settings, and the concerns raised are specific to nations without widespread access to specialized services, such as excisional lymph node biopsy. Lastly, due to the inherent limitations of this study, conclusions should be viewed as preliminary rather than definitive. 

## Conclusions

Histological confirmation of the etiology of lymphadenopathy is crucial in PLHA to differentiate between LPD and TB. Empiric TB treatment without appropriate confirmation of TB can lead to worse patient outcomes and significantly delay oncological care of PLHA. We recommend expanding biopsy access in resource-limited settings to prevent diagnostic delays and improve patient outcomes.
